# Ibuprofen Protects Ventilator-Induced Lung Injury by Downregulating Rho-Kinase Activity in Rats

**DOI:** 10.1155/2014/749097

**Published:** 2014-06-12

**Authors:** Liang-Ti Huang, Chien-Huang Lin, Hsiu-Chu Chou, Chung-Ming Chen

**Affiliations:** ^1^Graduate Institute of Clinical Medicine, College of Medicine, Taipei Medical University, 252 Wu-Hsing Street, Taipei 110, Taiwan; ^2^Department of Pediatrics, Wan Fang Hospital, Taipei Medical University, No. 111, Section 3, Hsing-Long Road, Taipei 116, Taiwan; ^3^Graduate Institute of Medical Sciences, College of Medicine, Taipei Medical University, 252 Wu-Hsing Street, Taipei 110, Taiwan; ^4^Department of Anatomy, School of Medicine, College of Medicine, Taipei Medical University, 252 Wu-Hsing Street, Taipei 110, Taiwan; ^5^Department of Pediatrics, Taipei Medical University Hospital, 252 Wu-Hsing Street, Taipei 110, Taiwan; ^6^Department of Pediatrics, School of Medicine, College of Medicine, Taipei Medical University, 252 Wu-Hsing Street, Taipei 110, Taiwan

## Abstract

*Background*. Ventilator-induced lung injury-(VILI-) induced endothelial permeability is regulated through the Rho-dependent signaling pathway. Ibuprofen inhibits Rho activation in animal models of spinal-cord injury and Alzheimer's disease. The study aims to investigate ibuprofen effects on high tidal volume associated VILI. *Methods*. Twenty-eight adult male Sprague-Dawley rats were randomized to receive a ventilation strategy with three different interventions for 2 h: (1) a high-volume zero-positive end-expiratory pressure (PEEP) (HVZP) group; (2) an HVZP + ibuprofen 15 mg/kg group; and (3) an HVZP + ibuprofen 30 mg/kg group. A fourth group without ventilation served as the control group. Rho-kinase activity was determined by ratio of phosphorylated ezrin, radixin, and moesin (p-ERM), substrates of Rho-kinase, to total ERM. VILI was characterized by increased pulmonary protein leak, wet-to-dry weight ratio, cytokines level, and Rho guanine nucleotide exchange factor (GEF-H1), RhoA activity, p-ERM/total ERM, and p-myosin light chain (MLC) protein expression. *Results*. Ibuprofen pretreatment significantly reduced the HVZP ventilation-induced increase in pulmonary protein leak, wet-to-dry weight ratio, bronchoalveolar lavage fluid interleukin-6 and RANTES levels, and lung GEF-H1, RhoA activity, p-ERM/total ERM, and p-MLC protein expression. *Conclusion*. Ibuprofen attenuated high tidal volume induced pulmonary endothelial hyperpermeability. This protective effect was associated with a reduced Rho-kinase activity.

## 1. Introduction

Mechanical ventilation has been used to support acutely ill patients for several decades. Despite the lifesaving potential of this treatment, it has several potential disadvantages and complications [1]. Mechanical ventilation with high tidal volumes causes lung edema and hemorrhage and activates inflammatory pathways, a process called ventilator-induced lung injury (VILI) [2]. Research has shown that the manifestations of VILI are physiologically and histopathologically indistinguishable from acute lung injury. The spectrum of VILI includes increased endothelial and epithelial permeability, increased pulmonary inflammatory mediators, and the formation of pulmonary edema [3, 4]. The ventilator-induced accumulation of neutrophil with myeloperoxidase activation has been suggested to play an important role in mediating acute inflammatory responses in VILI [5].

Pulmonary edema has been shown to be a prominent feature of VILI in small animal models and is the combination of impaired alveolar-capillary barrier integrity, increased alveolar fluid filtration, and decreased alveolar fluid clearance [6]. Edematous fluid is mostly produced by VILI-induced pulmonary endothelial hyperpermeability and lung barrier disruption. VILI-associated endothelial permeability is regulated through the Rho-dependent signaling pathway [7–9]. Activation of Rho signaling pathway may contribute to impaired endothelial function, promote actin stress fiber formation, and increase endothelial permeability [10]. In addition, the Rho/Rho kinase pathway has been shown to regulate the activation of ezrin, radixin, and moesin (ERM), which act as a link between several membrane receptors and the actin cytoskeleton, and to induce their phosphorylation and translocation to the apical cell membrane [11, 12]. The key members of Rho signaling pathway include Rho guanine nucleotide exchange factor H1 (GEF-H1), RhoA GDP, RhoA GTP, Rho-kinase (which may be presented as its subtract ERM protein), and myosin light chain (MLC) [13, 14]. Active GTP-bound RhoA can bind many effector proteins, including Rho-kinase involved in MLC phosphorylation, cytoskeletal dynamics regulation, and increase in endothelial permeability increment via microtubule depolymerization [14, 15].

Ibuprofen, a nonselective cyclooxygenase inhibitor, has been shown to act as a Rho activation inhibitor in animal models of spinal cord injury and Alzheimer's disease [16–19]. However, the* in vivo* effects of ibuprofen on the Rho signaling pathway in VILI are not well understood. The aims of this study were to test the hypotheses that high tidal volume ventilation-induced pulmonary edema involves the Rho-kinase activation and that ibuprofen pretreatment may attenuate pulmonary edema and inflammation by reducing the Rho-kinase activity in an animal model of VILI.

## 2. Material and Methods

### 2.1. Animal Preparation

The Animal Care and Use Committee of Taipei Medical University approved this study, which was performed with adult male Sprague-Dawley rats weighing 250–300 g.

### 2.2. Experimental Protocol

The rats were maintained on a 12 h light-dark cycle with free access to food and water. They were intraperitoneally anesthetized with pentobarbital (50 mg/kg, Abbott, North Chicago, IL, USA). Ibuprofen is rapidly metabolized and eliminated in the urine. Fifteen minutes before ventilation, the rats received either intravenous ibuprofen (15 mg/kg, *n* = 7; Pedea Orphan Europe SARL, Merckle GmbH, 10 mg/ampoule), ibuprofen (30 mg/kg; *n* = 7), or an equal volume of vehicle (*n* = 7) through the tail vein. The different ibuprofen dosage was based on recommendations by Daphtary et al. and Munro [20, 21]. We studied the dose-dependent anti-inflammatory effect of ibuprofen and later its effect on Rho-kinase activity in VILI. After tracheostomy, a 14 G plastic cannula was inserted into the trachea. The animals were then ventilated with a high-volume zero positive end-expiratory pressure (PEEP) (HVZP) protocol using a volume-cycled ventilator (Small Animal Ventilator, Model SAR-830/AP; CWE, Ardmore, PA, USA) for 2 h at a tidal volume of 35 mL/kg, a respiratory rate of 25 breaths/minute, and an FiO_2_ of 0.21. The rats were randomized to receive same ventilation strategy with different pharmacologic treatment. Another group received tracheostomy and no ventilation and served as a control group (*n* = 7). All animals were kept supine for the duration of the experiment.

After 2 h of ventilation, the rats were euthanized by intraperitoneal injections of pentobarbital (100 mg/kg), and their chest was opened and the lungs were removed intact with the tracheostomy tube in place. The right lung was ligated, and the left lung was lavaged with 2 mL 0.9% saline at 4°C. The saline was washed in and out of the lungs 3 times and then recovered. We repeated this washing procedure 2 more times for each animal, pooled the 3 washes, and recorded the total volume. There were no differences in the total volume of saline infused or recovered in this lavage procedure between the 4 experimental groups. An aliquot of the bronchoalveolar lavage fluid (BALF) from each animal was used to measure the total protein content with bovine serum albumin as the standard. The value was expressed as mg/kg body weight.

### 2.3. Lung Wet-to-Dry Weight Ratio

Lung wet-to-dry weight ratios were used as a measure of pulmonary edema. The fresh right lower lobe of each lung was weighed immediately after collection and placed into a 60°C oven to dry for 72 hours. The dried tissue was then weighed to determine the wet-to-dry weight ratio.

### 2.4. Bronchoalveolar Lavage Fluid Cytology, Myeloperoxidase, and Cytokines Analysis

We measured the BALF differential cell counts using light microscopy and determined the absolute cell numbers of macrophages, neutrophils, eosinophils, and lymphocytes. We used an enzyme-linked immunosorbent assay (ELISA) kit (R&D Systems, Minneapolis, MN, USA) to assay myeloperoxidase (MPO) and expressed the results as ng/mL lavage fluid. We assayed IL-1*β* and IL-6 using a commercially available ELISA kit (Innovative Research, Southfield, MI, USA) and used ELISA with Luminex Assays (High-throughput Multiplex Bead Based Assays, Panomics, CA, USA) to assay RANTES, the granulocyte macrophage colony-stimulating factor (GM-CSF), and interferon-gamma (IFN-*γ*).

### 2.5. Immunohistochemical Staining

Immunostaining was performed on paraffin sections with immunoperoxidase visualization. After the routine deparaffinization, heat-induced epitope retrieval was done by immersing the slides in 0.01 M sodium citrate buffer (pH 6.0). After blocking endogenous peroxidase activity and nonspecific binding of antibody, the sections were first preincubated for 1 h at room temperature in 0.1 M phosphate-buffered saline containing 10% normal goat serum and 0.3% H_2_O_2_ before being incubated for 20 h at 4°C with goat polyclonal anti-ERM (C-15) and rabbit polyclonal anti-RhoA (119) (1 : 100 dilutions; Santa Cruz Biotechnology Inc., Santa Cruz, CA, USA) were used as the primary antibodies. The sections were then treated for 1 h at room temperature with biotinylated rabbit anti-goat IgG for ERM and biotinylated goat anti-rabbit IgG for RhoA (1 : 200, Jackson Immunoresearch Laboratories Inc., PA, USA). Following reactions with the reagents from an ABC kit (Avidin-Biotin Complex, Vector, CA, USA) as per manufacturer's recommendations, the reaction products were visualized by 3,3 diaminobenzidine, 0.003% H_2_O_2_ in 0.5 M TRIS buffer, and pH 7.6 before the sections were mounted on gelatin-coated slides using Permount (Fisher, USA). All immunostained sections were viewed and photographed using a Nikon Eclipse E600 (Tokyo, Japan).

### 2.6. Western Blotting Analysis

Lung tissues were homogenized in a cold buffer containing 50 mM Tris-HCl (pH 7.5), 400 mM NaCl, 2 mM EGTA, 1 mM EDTA, 1 mM DTT, 10 *μ*M PMSF, 10 *μ*g mL-1 leupeptin, 1 *μ*g mL-1 pepstatin, and 1 mM benzamidine. Nuclei and unlysed cells were removed by low-speed centrifugation at 12000 ×g, at 4°C for 10 minutes. We determined the protein concentration of the supernatant using the Lowry method. The supernatant (200 *μ*g of protein) was mixed with an equal volume of a 2 × SDS sample buffer and boiled for 5 minutes. We separated proteins using SDS polyacrylamide gel electrophoresis (10% acrylamide) and blotted the sample onto a PVDF membrane. The membranes were blocked for 1 h with 5% (w/v) dry nonfat milk in TBS-tween. The blots were then incubated with rabbit polyclonal anti-RhoA (119), goat polyclonal antibody anti-GEF-H1 (Y-20), goat polyclonal anti-ERM, goat polyclonal anti-p-moesin (Thr 558) for p-ERM, which also binds to phosphorylated ezrin (Thr567) and radixin (Thr564), and goat polyclonal anti-p-MLC (Thr18/Ser19) (Santa Cruz Biotechnology Inc., Santa Cruz, CA, USA) for 20 h. Rho-kinase activity was determined by Western blot analysis of the ratio between the combined amount of T567-phosporylated ezrin, T564-phosphorylated radixin, and T558-phosphorylated moesin (p-ERM), and the total amount of ezrin, radixin, and moesin (ERM) (p-ERM/total ERM ratio) [22]. We used an HRP-conjugated secondary antibody in conjunction with an enhanced chemiluminescence detection kit from Amersham Pharmacia Biotech to visualize the immunopositive bands on X-ray film. We confirmed equal protein loading using an actin antibody and stained the membranes with Coomassie Brilliant Blue. We quantified the intensities of the bands using densitometry and a Scion image computer program software (Scion Corp. Beta 4.0.2). The densitometry unit of the protein expression in the control group was 1 after being normalized to *β*-actin.

### 2.7. RhoA Activity Assay

Active GTP-bound RhoA can be presented by RhoA activity assay. Rho activation pull-down assay was performed in the lysates collected from lung tissue using a RhoA activation assay Biochem kit (Cytoskeleton Inc., Denver, CO, USA) according to manufacturer's indications. Briefly, supernatants were incubated with Rhotekin Rho-binding domain at 4°C (×1 h) on a rotator. Bead-precipitated proteins were fractionated and immunoblotted with antibody against RhoA.

### 2.8. Histology

Immediately after the bronchoalveolar lavage was finished, right lung was fixed by instillation of a 10% formaldehyde solution at 20 cm H_2_O. Specimens were embedded in paraffin, stained with hematoxylin and eosin, and examined by a pathologist who was blinded to the protocol and experimental groups.

### 2.9. Electron Microscopy

The lungs were immersed and fixed in 4% paraformaldehyde in 0.1 M phosphate buffer. The lung tissues were then minced into 1 mm^3^ blocks. Ultrathin sections (50–60 nm) were double-stained with uranyl acetate and lead citrate and then examined using a Hitachi 600 electron microscope (Tokyo, Japan).

### 2.10. Statistical Analysis

The data were presented as the mean ± SD. We analyzed statistically significant differences using one-way ANOVA with the post hoc Bonferroni test and considered differences to be significant at a *P* value less than 0.05.

## 3. Results

### 3.1. Effects of Ibuprofen on Pulmonary Endothelial Permeability

**Figure 1:**
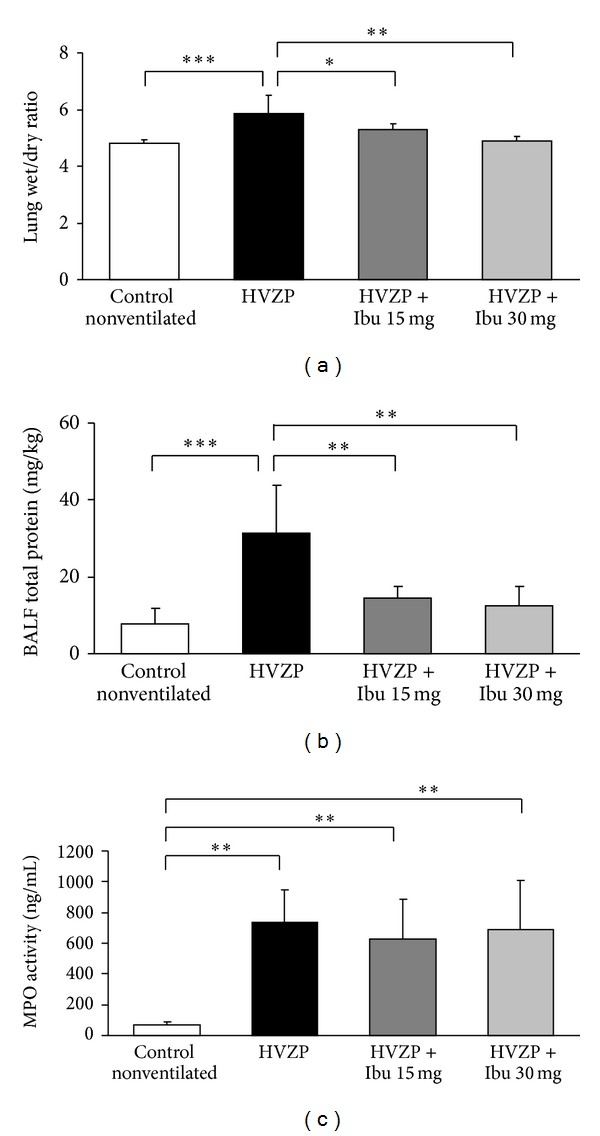
(a) Lung wet-to-dry weight ratio, (b) total protein concentration, and (c) myeloperoxidase (MPO) activity in bronchoalveolar lavage fluid (BALF) in the control, high volume zero pressure (HVZP), HVZP + ibuprofen (Ibu) 15 mg/kg, and HVZP + ibuprofen 30 mg/kg groups. All rats were randomly divided into four groups: the control group (*n* = 7) did not receive ventilation; the HVZP group (*n* = 7) received 2 hours of ventilation at a tidal volume of 35 mL/kg, a respiratory rate of 25 breaths/minute, and an FiO_2_ of 0.21; the HVZP + ibuprofen 15 mg/kg group (*n* = 7) received an intravenous injection of ibuprofen (15 mg/kg) 15 minutes before the HVZP ventilation; and the HVZP + ibuprofen 30 mg/kg group (*n* = 7) received an intravenous injection of ibuprofen (30 mg/kg). *P < 0.05, **P < 0.01, and ***P < 0.001.

The lung wet-to-dry weight ratio and total BALF protein content are the two measurements that are used to represent pulmonary edema and pulmonary endothelial permeability. In our animals, the wet-to-dry weight ratio of the lung tissue was significantly increased in the HVZP group when compared with the control group (Figure 1(a)). Interestingly, the administration of ibuprofen significantly reduced the lung wet-to-dry weight ratio at a dosage of either 15 mg/kg or 30 mg/kg. The total protein contents recovered from the BALF were significantly higher in rats ventilated with the HVZP protocol than in the control group (Figure 1(b)). Treatment with either 15 mg/kg or 30 mg/kg ibuprofen again significantly reduced the HVZP ventilation-induced increase in the BALF protein content.

### 3.2. Effect of Ibuprofen on Pulmonary Neutrophil Counts

The acute inflammation induced by VILI is first detected by lung neutrophil accumulation and by increased neutrophil associated MPO activity. In this study, rats in HVZP group exhibited significantly increased BALF neutrophil numbers when compared with those of controls as shown in Table 1. Neutrophil MPO activity was also significantly increased in HVZP rats as shown in Figure 1(c). However, ibuprofen-pretreated HVZP groups presented no differences in BALF neutrophil numbers and MPO activity from animals without ibuprofen pretreatment.

### 3.3. Effect of Ibuprofen on Pulmonary Cytokines

**Figure 2:**
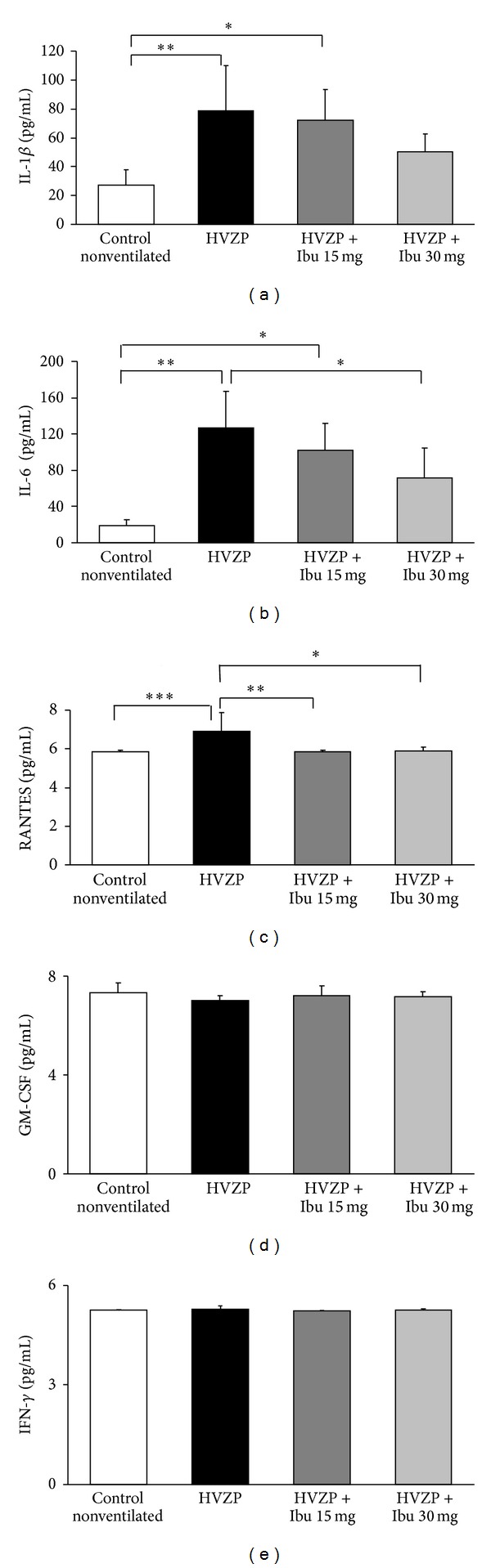
BALF (a) IL-1*β*, (b) IL-6, (c) RANTES, (d) GM-CSF, and (e) IFN-*γ* concentrations in the control, HVZP, HVZP + ibuprofen (Ibu) 15 mg/kg, and HVZP + ibuprofen 30 mg/kg groups. The treatment details are given in the legend of Figure 1. *P < 0.05, **P < 0.01, and ***P < 0.001.

The status of acute inflammation in our animals is then measured by BALF cytokines changes. The IL-1*β*, IL-6, and RANTES concentrations in the BALF significantly increased after HVZP ventilation, and the values of IL-1*β* and IL-6 were approximately 2- and 5-fold higher in the HVZP group than in the control group, respectively (Figures 2(a) and 2(b)). The administration of ibuprofen at 30 mg/kg significantly reduced the HVZP ventilation-induced increase in the BALF IL-6 (Figure 2(b)). The administration of ibuprofen at 15 mg/kg and 30 mg/kg also significantly reduced the HVZP ventilation-induced increase in the BALF RANTES (Figure 2(c)). The concentrations of GM-CSF and IFN-*γ* in BALF were comparable among the study groups (Figures 2(d) and 2(e)).

### 3.4. Effects of Ibuprofen on Total RhoA and ERM Expression and on Rho-Kinase Activity

**Figure 3:**
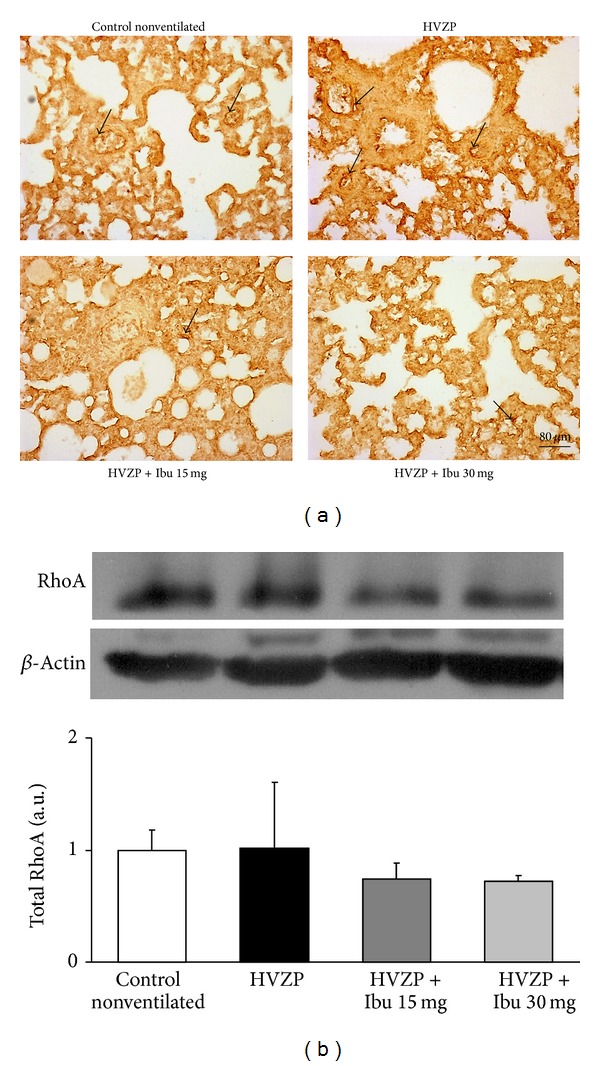
Lung total RhoA protein expression in the control, HVZP, HVZP + ibuprofen (Ibu) 15 mg/kg, and HVZP + ibuprofen 30 mg/kg groups. The treatment details are given in the legend of Figure 1 (*n* = 4 per group). (a) Lung immunohistochemistry showed dominant total RhoA expression on capillary endothelium (black arrow). (b) Bar graphs show the quantitative analysis of Western blot densitometry data.

**Figure 4:**
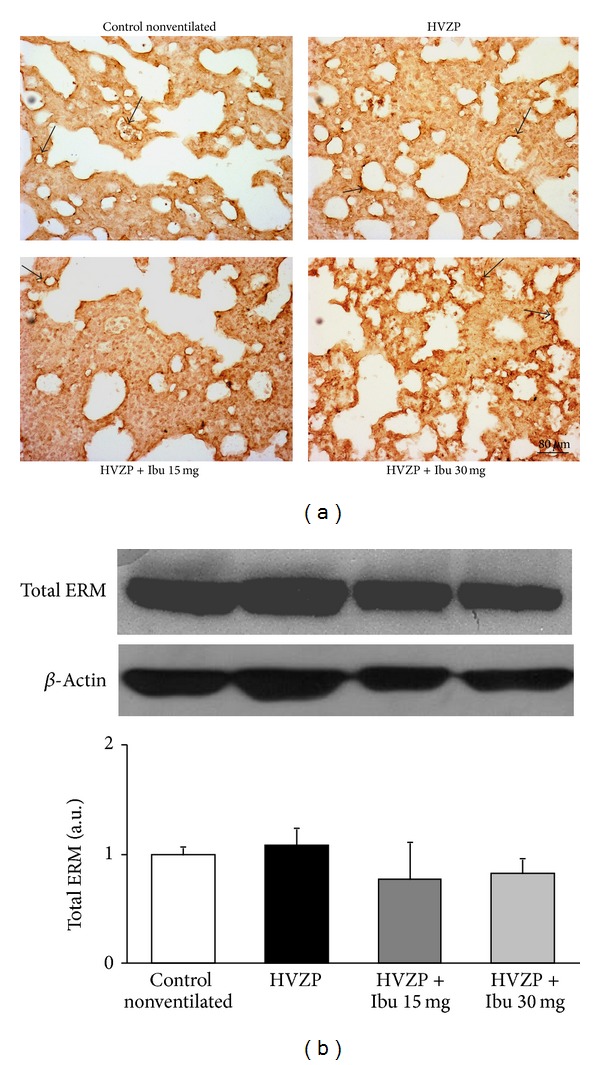
Lung total ERM protein expression in the control, HVZP, HVZP + ibuprofen (Ibu) 15 mg/kg, and HVZP + ibuprofen 30 mg/kg groups. The treatment details are given in the legend of Figure 1. (a) Lung immunohistochemistry showed dominant total ERM expression on capillary endothelium (black arrow). (b) Bar graphs show the quantitative analysis of Western blot densitometry data.

To investigate the mechanisms of VILI induced lung edema and the effects of ibuprofen pretreatment, we set to study the Rho pathway that may act on lung endothelial cells. Tissue distributions of total RhoA and its regulated ERM are first examined by immunohistochemistry. The lung immunohistochemistry showed predominant expression of total RhoA and total ERM in lung capillary endothelial cell in the control, HVZP, and ibuprofen pretreatment animals (Figures 3(a) and 4(a)). The quantitative measurement of total RhoA and total ERM expression is done by western blot and presented in Figures 3(b) and 4(b). The results showed that total RhoA and total ERM were maintained throughout treatments; protein levels were not changed after HVZP ventilation either with or without ibuprofen pretreatment when compared with the control.

**Figure 5:**
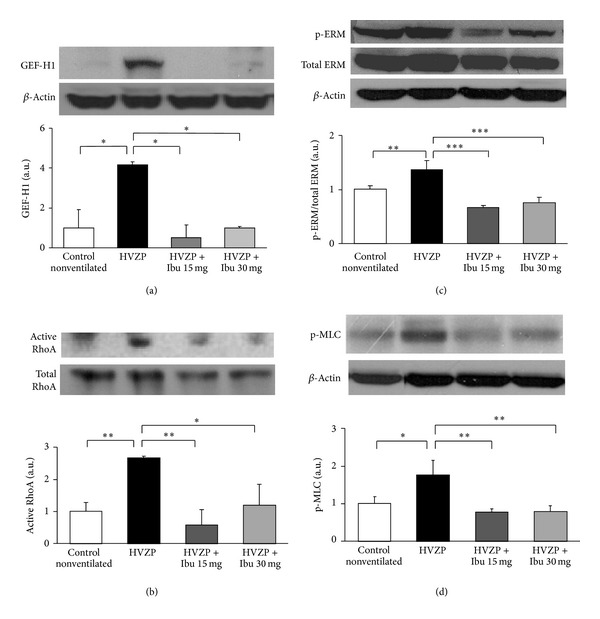
Lung (a) GEF-H1, (b) active RhoA, (c) p-ERM/total ERM, and (d) p-MLC protein expression in the control, HVZP, HVZP + ibuprofen (Ibu) 15 mg/kg, and HVZP + ibuprofen 30 mg/kg groups. The treatment details are given in the legend of Figure 1. *P < 0.05, **P < 0.01, and ***P < 0.001.

Rho-kinase is activated by active form of RhoA, which is catalyzed by GEF-H1 from inactive form of total RhoA. Since Rho/Rho kinase pathway leads to the activation of ERM, Rho-kinase activity can thus be determined by the p-ERM/total ERM ratio. One other activated RhoA-led pathway is to the MLC phosphorylation that ends with endothelial cell permeability regulation. In this study, the group that received 2 h of HVZP ventilation exhibited significant increases in the expression of GEF-H1, active RhoA, p-ERM/total ERM ratio, and p-MLC in the lung tissue when compared to the control and HVZP + ibuprofen groups (Figures 5(a)–5(d)). Pretreatment with ibuprofen significantly reduced the HVZP ventilation-induced increases in lung GEF-H1, active RhoA, p-ERM/total ERM ratio, and p-MLC expression.

### 3.5. Effects of Ibuprofen on Lung Histology

**Figure 6:**
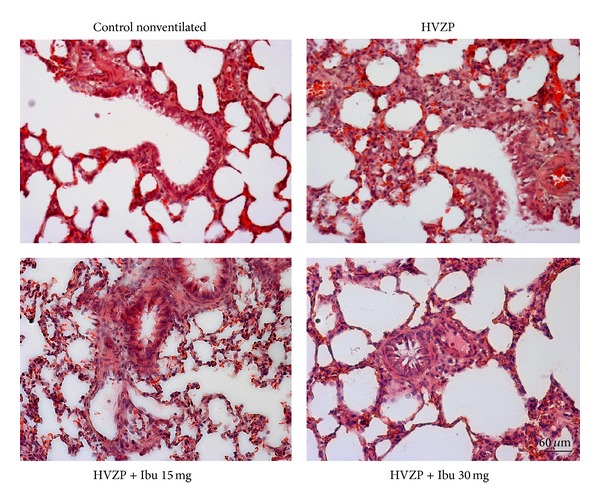
Representative lung histology in the control, HVZP, HVZP + ibuprofen (Ibu) 15 mg/kg, and HVZP + ibuprofen 30 mg/kg groups. The treatment details are given in the legend of Figure 1. Control group showing no major histologic abnormalities. HVZP group showing patchy areas of hemorrhage and thickened alveolar walls with inflammatory cells infiltration. HVZP + Ibu 15 mg/kg and HVZP + Ibu 30 mg/kg groups showing less hemorrhage and mild inflammatory cell infiltration.

Representative lung histology revealed that control group exhibited no major histologic abnormalities (Figure 6). HVZP group exhibited patchy areas of hemorrhage and thickened alveolar walls with inflammatory cells infiltration. Pretreatment with ibuprofen reduced lung injury.

### 3.6. Effects of Ibuprofen on Endothelial Ultrastructure

**Figure 7:**
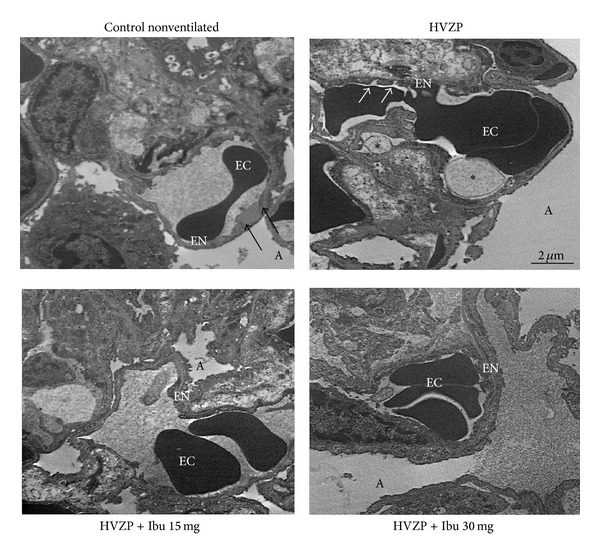
Representative electron micrographs of the control, HVZP, HVZP + ibuprofen (Ibu) 15 mg/kg, and HVZP + ibuprofen 30 mg/kg groups. The capillary profile was normal and is lined by continuous endothelium (black arrow). The HVZP group showed discontinuous endothelial cell lining (white arrow) and large endothelial cell membrane vacuolization (∗) protruding into the capillary lumen. The ultrastructure of the HVZP + Ibu 15 mg/kg and HVZP + Ibu 30 mg/kg groups revealed normal endothelial continuity as the control group. EC: erythrocyte, EN: endothelium, and A: alveolar space.

This study further used electron microscopy to investigate the ultrastructure changes of pulmonary alveoli during VILI and ibuprofen pretreatment. Electron microscopy of lungs showed normal ultrastructural appearance of alveolar capillaries in control rats (Figure 7) and a continuous layer of endothelial cells lined the capillaries. By contrast, alveolar capillaries in the HVZP group were destructed and presented discontinuous endothelial cell lining. The administration of ibuprofen showed protective effects on HVZP induced endothelial destruction.

## 4. Discussion

The results of the proposed* in vivo* lung injury model are consistent with alterations known to occur in VILI. The main findings of this study are that VILI is associated with pulmonary edema, increased levels of BALF inflammatory cytokines IL-1*β*, IL-6, and RANTES, neutrophil counts, MPO activity, lung Rho activation, and ultrastructural damage of alveolar endothelium. Ibuprofen pretreatment reduced the lung wet-to-dry weight ratio, BALF protein content, IL-6 and RANTES levels, lung GEF-H1, Rho activation, p-MLC expression, and alveolar endothelial injury. These results indicated that the increment of Rho-kinase activity related to high tidal volume ventilation-induced lung injury was suppressed by ibuprofen pretreatment.

Pulmonary edema is a prominent feature of VILI. The protein-rich content of the edema fluid suggests that pulmonary edema is due to increased pulmonary permeability implicating changes in the microvascular barriers. Ibuprofen had been used as a cyclooxygenase inhibitor to reduce ventilator-induced pulmonary edema in animal models [23, 24]. However, the mechanism that mediates the effect of ibuprofen on ventilation-induced vascular permeability remains unknown. Ibuprofen was reported to act as a Rho activity inhibitor in lowering amyloidogenic A*β*42 deposition in an animal model of Alzheimer's disease [16]. In the current study, we examined the effects of ibuprofen on lung microvascular changes and Rho-kinase activity in ventilator-induced pulmonary edema. Our results showed an obvious reduction of pulmonary endothelial permeability after ibuprofen pretreatment.

Lung total RhoA and total ERM protein expression were found dominantly on capillary endothelium. Lung GEF-H1, active RhoA, p-ERM, and p-MLC protein expressions increased during VILI and decreased in the ibuprofen-pretreated HVZP groups. These results demonstrate the location of the Rho-kinase pathway in the lung tissues and its downstream changes in VILI and after ibuprofen pretreatment. Previous studies have shown that the depletion of GEF-H1 or the expression of the dominant negative GEF-H1 mutant in human endothelial cells and in a murine model of VILI significantly attenuates endothelial permeability and vascular leakage induced by lung ventilation at a high tidal volume [25, 26]. RhoA activates several downstream permeability-increasing mediators such as thrombin or TNF-*α* which contributes to increased endothelial permeability [10]. In this study, the decreased GEF-H1 expression and RhoA activity after ibuprofen administration may explain the reduced pulmonary edema and endothelial permeability. Our study showed a comparable effect of the lower ibuprofen dosage (15 mg/kg) on downregulating GEF-H1 expression and RhoA activity. These findings are contrary to the observation of the dose-dependent effect of ibuprofen on BALF IL-6. The phosphorylation of ERM protein subsequently phosphorylates the regulatory subunit MYPT1, inhibits the MLC phosphatase, and enhances phosphorylation of MLC and stress fiber formation. This in turn stimulates cell contraction, endothelial permeability, and lung barrier disruption [27]. This study demonstrated that attenuated high tidal volume induced-pulmonary edema was associated with reduced lung GEF-H1, active RhoA, Rho-kinase activation (p-ERM/total ERM), and p-MLC expression in lung endothelial cells. These results suggest that activation of Rho-kinase plays an important role in the pathogenesis of VILI.

This study showed that ibuprofen pretreatment improved lung edema probably is not through decreased neutrophil infiltration and the ensued alterations of MPO activity. Previous studies have investigated neutrophil-mediated inflammatory processes in experimental models of VILI and found that neutrophils are predominant in the BALF obtained from animals ventilated with high tidal volume mechanical ventilation [28, 29]. MPO is a peroxidase enzyme that is most abundantly expressed in neutrophil granulocytes [30], and MPO activity reflects the neutrophil infiltration following local tissue or organ injury. Attenuation of MPO activity was shown to reduce the associated pulmonary neutrophil infiltration and improve lung histology in an isolated and perfused rat lung model [31]. The current study showed that high tidal volume ventilation was associated with a significant increase in the BALF neutrophil counts and MPO activity and that ibuprofen pretreatment did not suppress these effects. The negligible effects of ibuprofen on BALF MPO activity are similar to an animal model of endotoxin-induced lung injury [20].

The current study showed that the BALF IL-1*β*, IL-6, and RANTES concentrations increased after high tidal volume ventilation. Pretreatment with ibuprofen at dosages of 15 mg/kg and 30 mg/kg significantly reduced the VILI-induced elevation of the RANTES level in the BALF, and at a dosage of 30 mg/kg, ibuprofen reduced the VILI-associated BALF concentration of IL-6. However, neither dosage of ibuprofen could reduce BALF IL-1*β* concentration. The expression of RANTES in the airway has been shown to increase in ischemia reperfusion-induced lung injury and respiratory virus infection [32]. Growth factors such as GM-CSF and IFN-*γ* drive the differentiation and activation of macrophage progenitors or lineage precursors [33]. The current study found that GM-CSF and IFN-*γ* concentrations remained unchanged after VILI and that ibuprofen administration did not influence the concentrations of GM-CSF or IFN-*γ*. These results may explain the BALF macrophage cell numbers in this study and are in agreement with the results of Niitsu et al. who found that pretreatment with ibuprofen effectively attenuated lung edema without affecting the ventilator-induced activation of inflammatory cytokines (tumor necrosis factor-*β* and IL-1*β*) [23].

There were two limitations in this study. The main limitation is that less aggressive low tidal volume (6–8 mL/kg) ventilation was not applied to evaluate its relevance to ibuprofen and Rho-kinase activity. While a nonventilated group may be appropriate to help identify the effects of mechanical ventilation per se, a group receiving less aggressive ventilation (pretreated with or without ibuprofen) would have been interesting too. Low tidal volume ventilation has been shown to increase lung inflammatory cytokine without significant edema formation [34]. Data on Rho-kinase activity and treatment effects of ibuprofen in such a setting would have been interesting. This study aims to show the beneficial effects of ibuprofen on Rho-kinase activity and pulmonary edema in injurious ventilation. Therefore, low tidal volume strategy was not used in this study. The second limitation is that the hemodynamic variables were not measured in this animal model. Ibuprofen has been shown to improve heart rate and blood pressure during endotoxic shock in rabbits [35]. The effect of ibuprofen on hemodynamic variables in VILI was unknown. Although we did not monitor hemodynamic variables, none of the animals died throughout the ventilation period. We cannot exclude the beneficial effects of ibuprofen on hemodynamic functions in this study.

## 5. Conclusion

This study showed that high tidal volume ventilation increased total protein, IL-1*β*, IL-6, RANTES, neutrophil counts, and MPO activity in the BALF and induced lung edema, GEF-H1 and active RhoA expression, Rho-kinase activation, and ultrastructural evidence of endothelial destruction. Ibuprofen pretreatment reduced the BALF RANTES, IL-6 levels, and pulmonary endothelial destruction by attenuation of lung tissue GEF-H1 expression and RhoA/Rho-kinase activity. These findings demonstrated that ibuprofen protects high tidal volume induced pulmonary endothelial hyperpermeability by downregulating RhoA/Rho-kinase activity.

## Figures and Tables

**Table 1:** The number of cells in the BALF.

Treatment	*n*	Macrophage	Neutrophil	Eosinophil	Lymphocyte
Control	7	0.32 ± 0.30	0.01 ± 0.02	0.14 ± 0.10	4.30 ± 3.70
HVZP	7	1.47 ± 1.27	1.10 ± 0.67**	0.43 ± 0.47	13.6 ± 15.8
HVZP + Ibu 15 mg/kg	7	0.91 ± 0.71	0.75 ± 0.42	0.25 ± 0.10	7.50 ± 5.30
HVZP + Ibu 30 mg/kg	7	0.33 ± 0.10	0.95 ± 0.67	0.10 ± 0.20	5.20 ± 2.80

Cell numbers were measured as 10^4^ cells/mL of macrophages, neutrophils, eosinophils, and lymphocytes, and the values are expressed as mean ± SD. BALF: bronchoalveolar lavage; HVZP: high volume zero pressure ventilation. **P < 0.01 compared to control group.
